# IL-10-Producing CD1d^hi^CD5^+^ Regulatory B Cells May Play a Critical Role in Modulating Immune Homeostasis in Silicosis Patients

**DOI:** 10.3389/fimmu.2017.00110

**Published:** 2017-02-13

**Authors:** Ying Chen, Chao Li, Yiping Lu, Huiying Zhuang, Weijia Gu, Bo Liu, Fangwei Liu, Jinkai Sun, Bo Yan, Dong Weng, Jie Chen

**Affiliations:** ^1^Division of Pneumoconiosis, School of Public Health, China Medical University, Shenyang, China; ^2^Department of Respiratory Medicine, Shenyang No. 9 Hospital, Shenyang, China; ^3^Department of Respiratory Medicine, Shanghai Pulmonary Hospital, Tongji University School of Medicine, Shanghai, China

**Keywords:** IL-10, regulatory B cells, regulatory T cells, Th1/Th2 polarization, immune homeostasis, silicosis patients

## Abstract

Silicosis is characterized by chronic lung inflammation and fibrosis, which are extremely harmful to human health. The pathogenesis of silicosis involves uncontrolled immune processes. Evidence supports that regulatory B cells (Bregs) produce negative regulatory cytokines, such as IL-10, which can negatively regulate immune responses in inflammation and autoimmune diseases. Our previous study found that IL-10-producing B cells were involved in the development of silica-induced lung inflammation and fibrosis of mice. However, little is known about the role of Bregs in silicosis patients (SP). In this study, we found that serum concentrations of IL-10 were significantly increased in SP by using protein array screening. We further determined that the frequency of IL-10-producing CD1d^hi^CD5^+^ Bregs, not IL-10-producing non-B lymphocytes, was significantly higher in SP compared to subjects under surveillance (SS) and healthy workers (HW) by flow cytometry. We also found that regulatory T cells (Tregs) and Th2 cytokines (IL-4, IL-5, and IL-13) were significantly increased in SP. Th1 cytokines (IFN-γ, IL-2, and IL-12) and inflammatory cytokines (IL-1β, IL-6, and TNF-α) were not significantly different between SP, SS, and HW. Our study indicated that IL-10-producing CD1d^hi^CD5^+^ Bregs might maintain Tregs and regulate Th1/Th2 polarization in SP, suggesting that IL-10-producing Bregs may play a critical role in modulating immune homeostasis in SP.

## Introduction

Silicosis is a potentially fatal, but preventable, occupational lung disease caused by inhaling respirable crystalline silica (1). It is characterized by chronic lung inflammation and irreversible fibrosis (2). Progressive fibrosis leads to disability and mortality in patients, which results in a heavy burden to society. In China, there were 11,471 cases of silicosis reported in 2014, accounting for 42.69% of 26,873 new pneumoconiosis cases (Ministry of Health of the People’s Republic of China, 2015). Silicosis is reported to be a special concern in young adults (aged 15–44 years) (3). Silicosis has become the most serious occupational disease in both developed and developing countries, yet little is known about the crucial cellular and molecular mechanisms that initiate and propagate the process of silicosis.

Previous evidence has shown that both innate and adaptive immune responses play a key role in the pathogenesis of silicosis (4, 5). Alveolar macrophages, the first defense against foreign substances, ingest inhaled silica and become activated to release a host of mediators, such as cytokines and chemokines, which initiate the influx of inflammatory cells and activation of immune cells (6, 7). The complex network of interactions between mediators and cells results in the onset of lung injury, inflammation, and potentially fibrosis. The inflammation mediators are involved in the causation of silicosis. Chemokines that primarily attract monocytes and macrophages induce inflammation mediators such as IL-1β, IL-6, and TNF-α, which initiate the activation of T and B immune cells and stimulate T-lymphocyte type switching (8, 9).

Previous studies have demonstrated that Th1 and Th2 cells participate in the pathogenesis of silicosis (10, 11). Regulatory T cells (Tregs) play a role of the maintenance of immune homeostasis, which limits inflammation and modulates Th1/Th2 balance in silica-induced lung fibrosis (12). However, other regulatory subsets in silicosis have not been investigated in detail, and the mechanism of immune homeostasis modulation needs further exploration. Recent studies have demonstrated that another regulatory lymphocyte, specific B cell subsets, so-called regulatory B cells (Bregs), are potent immune response regulators and play important roles in regulating immune homeostasis (13, 14). A number of Breg subsets have been reported, of which the IL-10-producing B cell (B10) subset is among the best characterized (15, 16). Subsequent studies have shown that CD1d^high^CD5^+^ B cells were associated with the suppression of inflammation and autoimmune disease through IL-10 secretion (17, 18). B10 was also found to participate in regulating Th immune response by affecting the secretion of inflammatory cytokines such as TNF-α (19–21). In addition, B10 influences the proliferation of Tregs (22). Recent findings have shown that frequencies of peripheral Bregs increased in patients with lung cancer and B10s were significantly elevated in non-small cell lung carcinoma patients (23, 24). CD1d^+^CD5^+^ B cell numbers were increased in patients with tuberculosis (25). Our previous study found that B10 was involved in the development of silica-induced lung inflammation and fibrosis of mice (26). However, little is known about the role of similar or equivalent regulatory B lymphocyte populations in silicosis patients (SP).

A protein microarray assay and Bio-Plex assay were used in the present study to find that the level of IL-10 was increased in the serum of SP. To explore whether the increased IL-10 was produced by B and/or T cell populations and to determine the role of Bregs in SP, we investigated peripheral B10 and CD19^+^ IL-10^+^CD1d^hi^CD5^+^ B cell subsets and Tregs and also determined the level of chemokines, inflammation mediators, and Th1 and Th2 cytokines in SP, subjects under surveillance (SS), and healthy workers (HW) that had prior exposure to silica dust. Our results may provide new insights into the role of Bregs in SP.

## Materials and Methods

### Study Design

The study, conducted from May 2015 to March 2016, included 57 individuals. Nineteen first phase male SP were recruited from Shenyang No. 9 Hospital. Nineteen SS and nineteen HW with exposure to silica dust from Northern Heavy Industries Group Co., Ltd., matched by sex, age, year of exposure to silica dust, and ethnicity with SP were enrolled in this study. Criteria were based on the national diagnostic criteria of pneumoconiosis (GBZ70-2009). SS were workers with exposure to silica, who had no certain shadow changes visible in the chest X-ray films by health examination, and who needed periodic follow-ups within specified periods of time. The diagnosis of silicosis was based upon patient history of exposure to silica and available chest X-rays and was made by five qualified experts, who served as members of the Shenyang Municipal Pneumoconiosis Diagnosis Committee. Patients with chronic inflammatory diseases, respiratory infectious diseases, tuberculosis, asthma, diabetes, or cancer autoimmune disease were excluded.

Pulmonary function was measured from 2015 to 2016 by using spirometry. The data collected included forced vital capacity (FVC) and forced expiratory volume in 1 s (FEV_1_). Personal information, including sex, age, exposure period, and smoking status were obtain *via* questionnaire.

This study was carried out in accordance with the recommendations of the Ethics Committee of the China Medical University with written informed consent from all subjects. All subjects gave written informed consent in accordance with the Declaration of Helsinki. The protocol (CMU2100012006) was approved by the Ethics Committee of the China Medical University.

### Blood Sample

Five milliliters of venous blood samples were collected into no additive vacutainer tubes. Serum was obtained after centrifugation and stored at −80°C for protein microarray analysis and the Bio-Plex assay.

Also, 6 ml venous blood samples were collected into vacutainer tubes containing EDTA. Peripheral blood mononuclear cells (PBMCs) were immediately isolated by Ficoll density gradient centrifugation (TBD sciences, China) for Flow cytometric analysis.

### Protein Microarray Analysis

RayBiotech Human Cytokine Antibody Array 440 (RayBiotech, Inc., Cat# QAH-CAA-440) was used to profile the variation of proteins in serum. The samples were detected by the Shanghai XY Biotech Company. The experimental procedure was carried out in accordance with the manufacturer’s instructions. Briefly, pre-coated antibody array membranes were incubated with coating buffer for 30 min. After that, the blocking buffer was decanted and replaced with sample dilutions. Membranes were incubated overnight at 4°C with shaking. The next day, the membranes were incubated with biotin-conjugated antibody for 2 h after washing. Mixture of biotin-conjugated antibodies was removed and Streptavidin-Fluor was added to each sub-array. The incubation chambers were covered with adhesive film and incubated for 2 h. After washing, signals were detected with a GenePix 4000B system (Axon Instruments, Foster City, CA, USA). GenePix Pro 6.0 software (Axon Instruments) was used for densitometric analysis of the spots. These values were normalized to the ratio of positive control values for each sample. Afterward, the total normalized fluorescence values of replicate spots were averaged and expressed as fold increase over the control samples.

### Bio-Plex Assay

The levels of 27 cytokines in serum were measured by the Bio-Plex Pro™ human cytokine 27-plex assay (Bio-Rad Laboratories, Hercules, CA, USA; Cat# M500KCAF0Y). This multiplex assay detects: FGF basic, Eotaxin, G-CSF, GM-CSF, IFN-γ, IL-1β, IL-1ra, IL-2, IL-4, IL-5, IL-6, IL-7, IL-8, IL-9, IL-10, IL-12 (p70), IL-13, IL-15, IL-17, inducible protein-10 (IP-10), monocyte chemoattractant protein-1 (MCP-1), macrophage inflammatory protein-1 (MIP-1)α, MIP-1β, PDGF-BB, RANTES, TNF-α, and VEGF. The experimental procedure was carried out according to the manufacturer’s instructions. Briefly, beads coated with capture antibodies were incubated with premixed standards or sample supernatants for 30 min. Following incubation, premixed detection antibodies were added and incubated as before. After washing, streptavidin-PE was added and incubated for 10 min. After washing, the beads were resuspended in Bio-Plex cytokine assay buffer, and results were read on the Bio-Plex 200 system using Low PMT setting. Data were analyzed with Bio-Plex Manager™ software version 2.0.

### Cell Preparation and Flow Cytometry Analysis

Peripheral blood mononuclear cells were isolated from blood samples by Ficoll density gradient centrifugation. After washing and addition of RPMI1640 medium, PBMCs were stimulated with Leukocyte Activation Cocktail (BD Pharmingen, San Jose, CA, USA) for 5 h, followed by blocking with Human BD Fc Block™ (BD Pharmingen) for 10 min at room temperature. PE-Cy7-conjugated CD4 (BD Pharmingen) and PerCP-Cy5.5-conjugated CD19 (BD Pharmingen) antibodies were used for cell surface staining. Cells were fixed and permeabilized using a fixation/permeabilization kit (eBioscience, San Diego, CA, USA) or BD Cytofix/Cytoperm™ Fixation/Permeabilization Solution kit (BD Pharmingen) according to the manufacturer’s instructions. The cells were then stained at 4°C with APC-conjugated anti-Foxp3 (BD Pharmingen), APC-conjugated anti-IL-10 (BD Pharmingen), FITC-conjugated anti-CD5 (BD Pharmingen), and PE-conjugated anti-CD1d (BD Pharmingen). After washing, stained cells were resuspended in 1% paraformaldehyde-PBS. Analysis of cell marker expression was performed using a FACSCanto II system (BD, Franklin Lakes, NJ, USA). Dead cells were gated out depending on forward scattering and side scattering. Cells were analyzed with FlowJo X software (Tree Star, OR, USA).

### Statistical Analyses

Data were analyzed for statistical significance using SPSS 19.0 software (SPSS Inc., Chicago, IL, USA). Means between two groups were analyzed by Student’s *t*-test. The differences between values were evaluated through a one-way analysis of variance followed by pair-wise comparison with the Student–Newman–Keuls test. The chi-square test was used to compare categorical variables. Also, *p* < 0.05 was considered statistically significant. All values are means ± SEM.

## Results

### Study Subjects

The general characteristics of the study subjects are shown in Table 1. No difference in general characteristics, including median age, exposure period, and smoking status, were found between three groups. Pulmonary function (%FVC predicted, %FEV_1_ predicted, %FEV_1_/FVC radio) in SP was significantly decreased compared to SS and HW.

**Table 1:** **Characteristics of study participants**.

	HW (male, *n* = 19)	SS (male, *n* = 19)	SP (male, *n* = 19)	*p*-Value
Age (years)	52.26 ± 0.82	52.32 ± 0.69	53.26 ± 1.00	0.65^a^
Exposure period (years)	30.21 ± 0.83	30.00 ± 0.92	28.05 ± 0.93	0.18^a^
%FVC predicted	90.81 ± 2.50	91.28 ± 3.46	67.41 ± 6.82#,*	0.001^a^
%FEV_1_ predicted	90.79 ± 3.15	95.45 ± 3.83	72.23 ± 6.20^#,^*	0.002^a^
%FEV_1_/FVC radio	84.90 ± 1.55	88.88 ± 1.49	71.91 ± 3.21^#,^*	<0.001^a^
Smoking, *N* (%)				0.94^b^
Never	7 (36.8)	8 (42.1)	8 (42.1)	
Past	1 (5.3)	1 (5.3)	2 (10.5)	
Current	11 (57.9)	10 (52.6)	9 (47.4)	

*HW, healthy workers with exposure to silica dust; SS, subjects under surveillance; SP, silicosis patients; FVC, forced vital capacity; FEV_1_, forced expiratory volume in 1 s*.

Values are expressed as means ± SEM, ^#^p < 0.05 compared with HW, *p < 0.05 compared with SS.

*^a^Calculated by one-way analysis of variance*.

*^b^Calculated by chi-square test*.

### Multiple Cytokines Altered in Serum of SP

**Figure 1:**
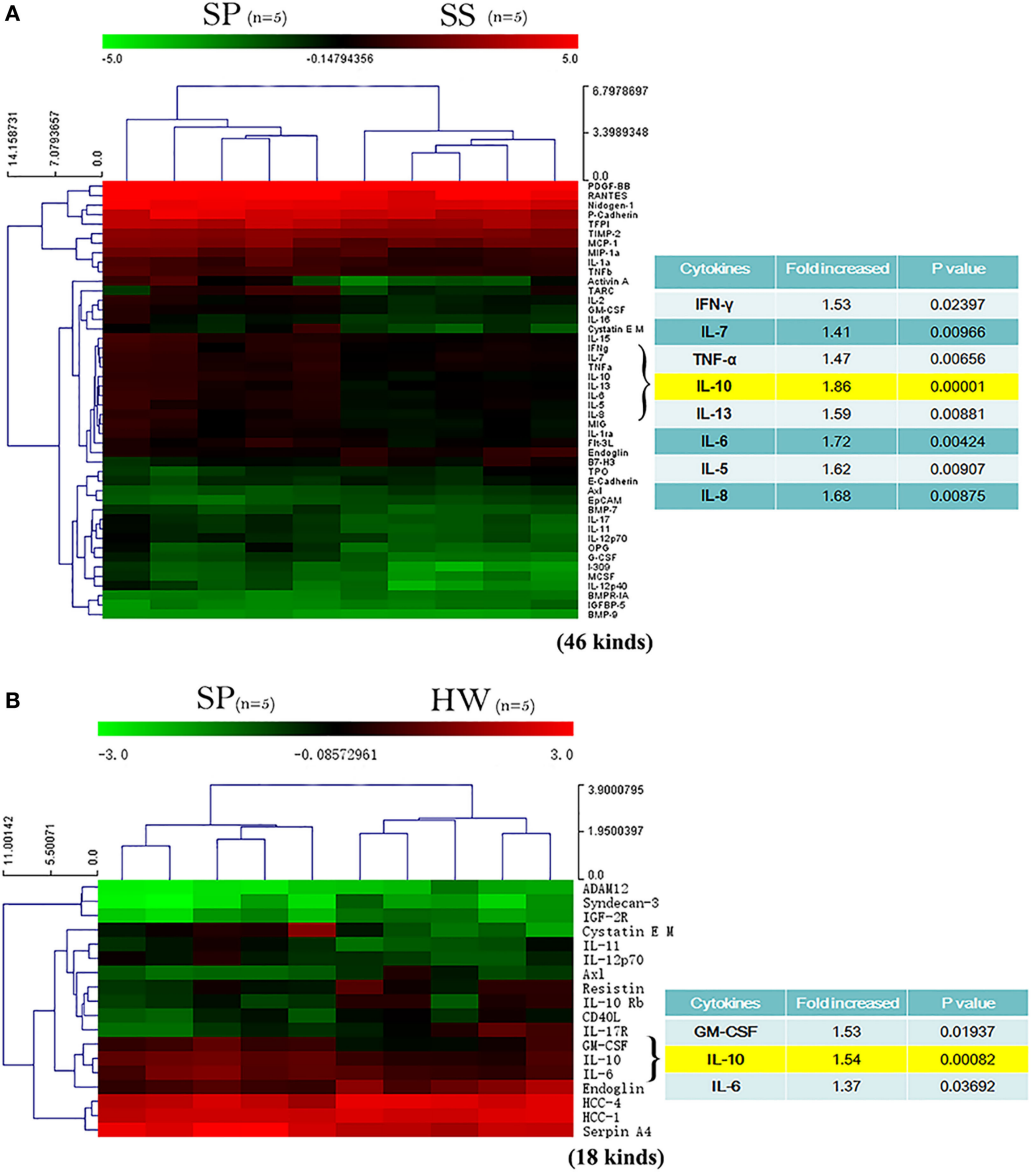
**A profile of cytokines in serum of silicosis patients (SP) was detected by protein microarray**. **(A)** Heat map analysis between SP and SS. Image showed a marked increase of IFN-γ, IL-7, TNF-α, IL-10, IL-13, IL-6, IL-5, and IL-8 in SP (*n* = 5) compared to SS (*n* = 5). Red represents upregulated protein expression, and green represents downregulated protein expression. **(B)** Heat map analysis between SP and workers exposed to silica dust. Image showed a marked increase of GM-CSF, IL-10, and IL-6 in SP (*n* = 5) compared to healthy workers (HW) (*n* = 5). Red represents upregulated protein expression, and green represents downregulated protein expression.

To profile the cytokines in the peripheral blood from patients with silicosis, a blinded screening using serum of SP, SS, and HW by protein microarray analysis was performed. As shown in the heat maps, 46 kinds of cytokines were different between SP and SS, and 18 kinds of cytokines were different between SP and HW. Fifty-six kinds of cytokines were different among HW, SS, and SP groups (data shown in Tables S1–S3 in Supplementary Material, respectively). Among those cytokines, there was a marked increase of IFN-γ, IL-7, TNF-α, IL-10, IL-13, IL-6, IL-5, and IL-8 in SP (*n* = 5) compared to SS (*n* = 5) (Figure 1A). There was also a marked increase of GM-CSF, IL-10, and IL-6 in SP (*n* = 5) compared to HW (*n* = 5) (Figure 1B). The levels of IL-10 showed significance difference between SP and SS (*p* = 0.00001). The levels of IL-10 were significantly different between SP and HW (*p* = 0.00082). These results suggested that IL-10 might play a critical role in the pathogenesis of silicosis.

### Serum Level of IL-10 and IL-10-Producing CD1d^high^CD5^+^ Bregs Were Increased in SP

**Figure 2:**
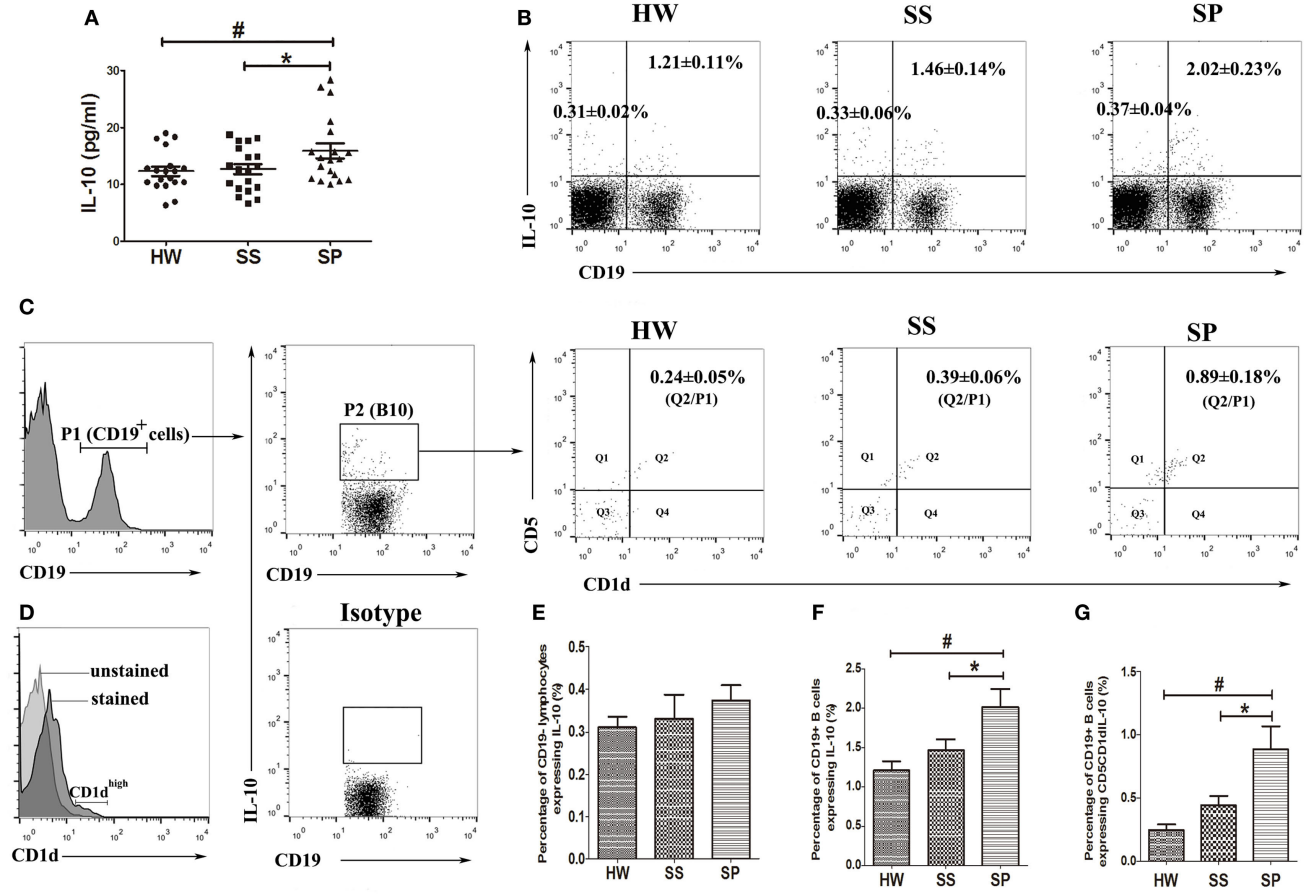
**Level of IL-10 in serum, IL-10-producing B cell (B10), and IL-10^+^CD1d^high^CD5^+^ B cell subsets in the peripheral blood were assayed in all participants**. **(A)** The level of IL-10 in serum was assayed by the Bio-Plex assay. **(B,E,F)** Percentages of CD19^+^IL-10^+^ B cells and CD19^-^IL-10^+^ cells in the peripheral blood were assayed by flow cytometry. **(C,G)** Percentage of IL-10^+^CD1d^high^CD5^+^ B cells in the peripheral blood was assayed by flow cytometry. **(D)** The overlay graph of CD1d unstained and CD1d stained histograms [*n* = 19 per group, ^#^*p* < 0.05 compared with healthy workers (HW); *p < 0.05 compared with SS]. Error bars indicate mean ± SEM.

The sample size was enlarged (*n* = 19), and the Bio-Plex array was used to further confirm the variation of serum IL-10 in SP. The Bio-Plex suspension array system permitted the simultaneous measurement of 27 human cytokines, including interleukins, chemokines, colony-stimulating factors, growth factors, interferon, and tumor necrosis factor (data of 27 cytokines shown in Table S4 in Supplementary Material). The results showed that the serum concentrations of IL-10 were significantly increased in SP compared to SS and HW (*p* = 0.03, *p* = 0.017) (Figure 2A). To explore the possibility that the increased IL-10 was produced by B cell and/or T cell populations, peripheral B10s and IL-10-producing non-B lymphocytes were investigated from all participants. The percentage of CD19^+^IL-10^+^ B cells was observed to significantly increase in SP (2.02 ± 0.23%) compared to the SS group (1.46 ± 0.14%) and the HW group (1.21 ± 0.11%) (Figures 2B,F). However, there were no obvious differences in the percentage of CD19^-^IL-10^+^ lymphocytes between the three groups (Figures 2B,E). These data suggested that increased serum IL-10 in SP might contribute to B10s, but not IL-10-producing non-B lymphocytes.

Emerging evidence indicates that CD1d^hi^CD5^+^ B cells are responsible for most of the IL-10 production by B cells (17). We investigated peripheral IL-10-producing CD1d^hi^CD5^+^ B cells in all participants. Our flow cytometry results showed that a proportion of cells highly expressed CD1d in CD19^+^ B cells (Figure 2D). The percentage of IL-10^+^CD1d^hi^CD5^+^ B cells significantly increased in SP (0.89 ± 0.18%) compared to the SS (0.39 ± 0.06%) and HW (0.24 ± 0.05%) groups (Figures 2C,G). These data suggested that IL-10^+^CD1d^hi^CD5^+^ B cells might play a critical role in the pathogenesis of silicosis.

### Serum Levels of Chemokines Were Increased in SS

**Figure 3:**
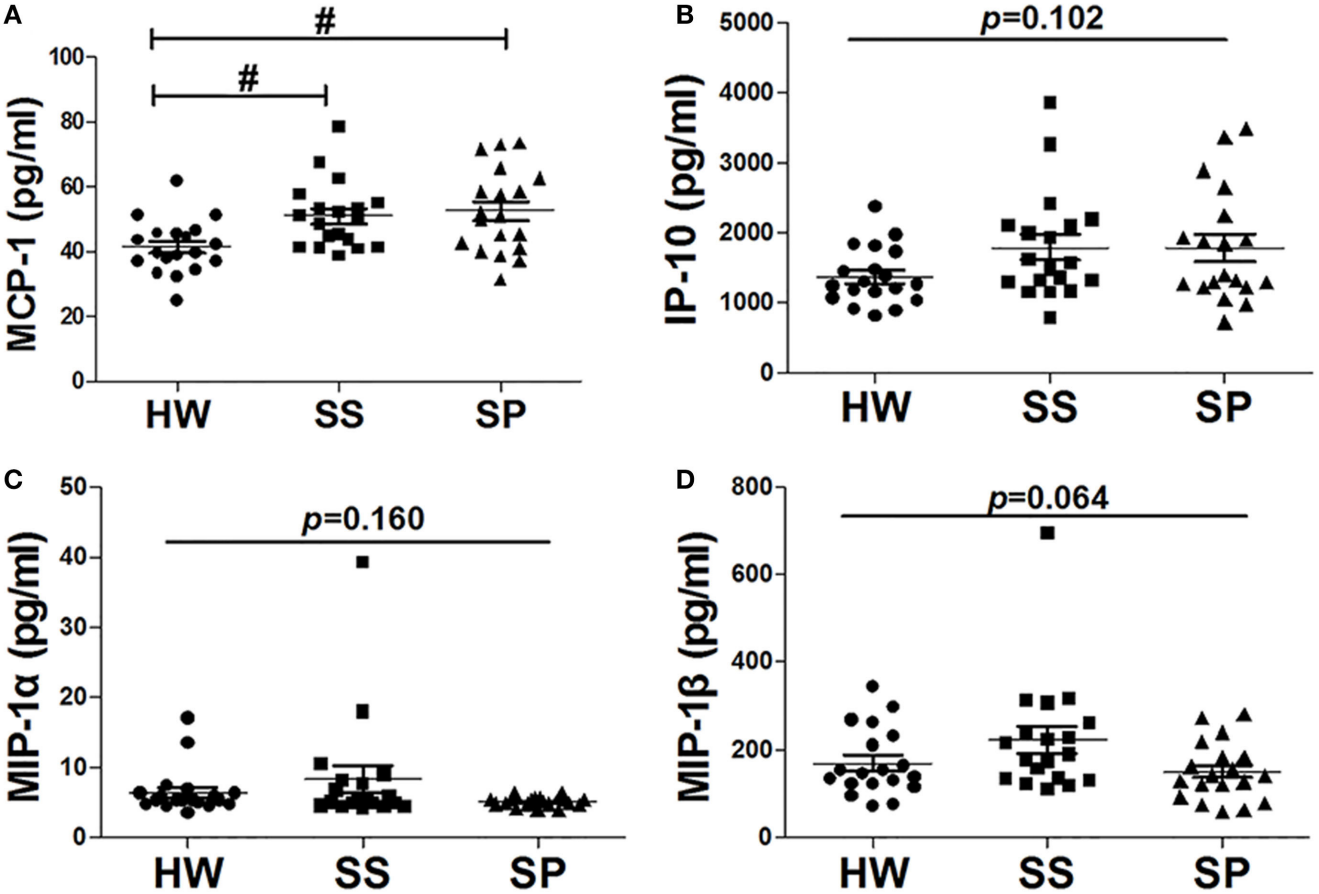
**Chemokines in serum were detected in all participants**. **(A)** Level of monocyte chemoattractant protein-1 (MCP-1) in serum was assayed by the Bio-Plex assay. **(B)** The level of inducible protein-10 (IP-10) in serum was assayed by the Bio-Plex assay. **(C)** The level of macrophage inflammatory protein-1 (MIP-1)α in serum was assayed by the Bio-Plex assay. **(D)** The level of MIP-1β in serum was assayed by the Bio-Plex assay [*n* = 19 per group, ^#^*p* < 0.05 compared with healthy workers (HW); *p < 0.05 compared with subjects under surveillance (SS)]. Error bars indicate mean ± SEM.

Of the 27 cytokines detected by the Bio-Plex assay, some chemokines tended to increase in the early stages of silicosis. Compared with HW, the serum concentration of MCP-1 in SS and SP was significantly increased (*p* = 0.008, *p* = 0.002) (Figure 3A). In addition, the serum concentration of interferon gamma-IP-10 was increased in SS and SP compared to HW (Figure 3B). The serum concentrations of MIP-1α and MIP-1β were increased in SS compared to HW and SP (Figures 3C,D), but these differences were not statistically significant. These results suggested that some chemokines might initially be secreted by activated macrophages.

### Tregs, Not Inflammatory Cytokines, Were Increased in SP

**Figure 4:**
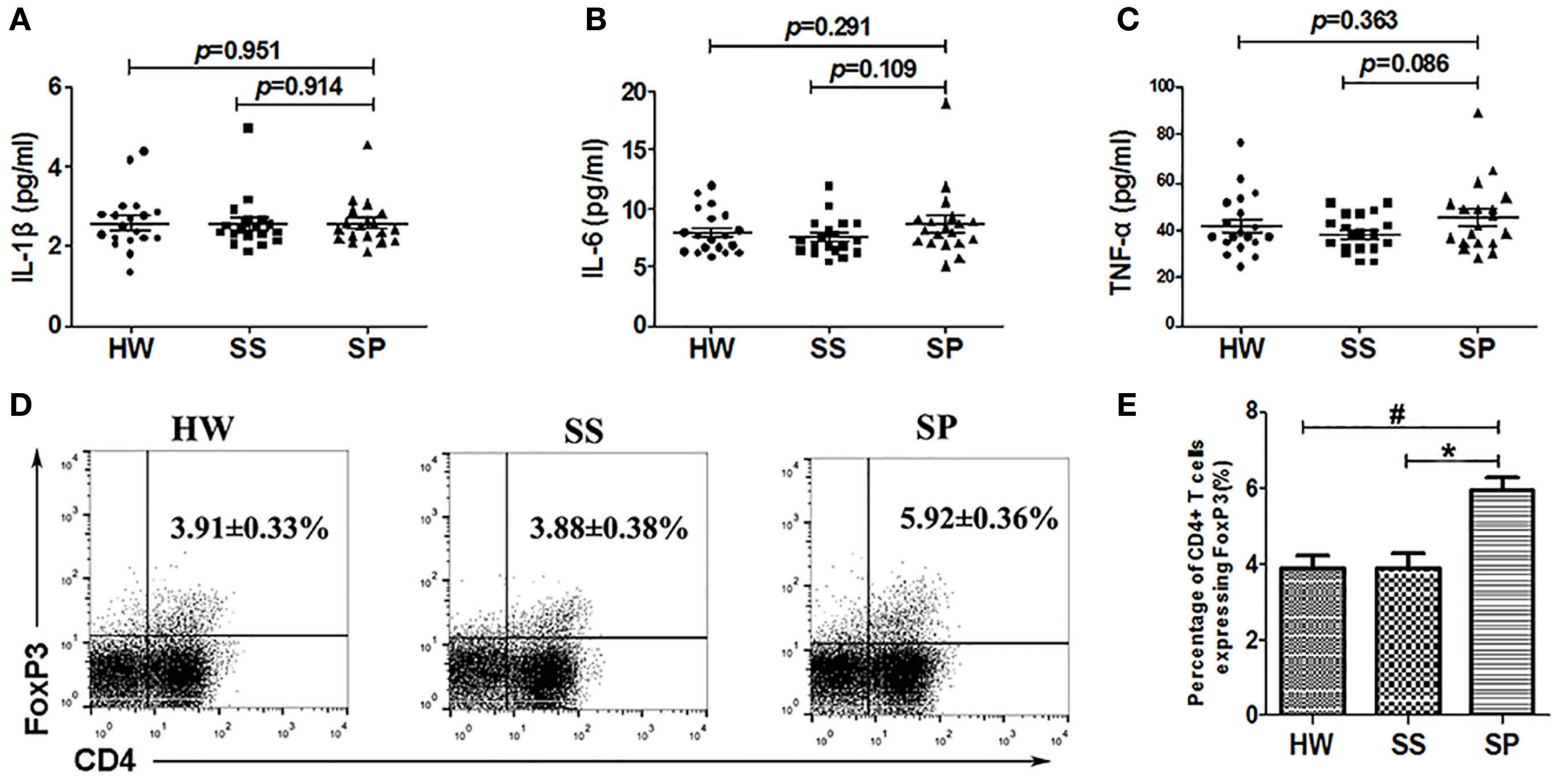
**Levels of inflammatory cytokines in serum and regulatory T cells (Tregs) in the peripheral blood were assayed in all participants**. **(A)** The level of IL-1β in serum was assayed by the Bio-Plex assay. **(B)** The level of IL-6 in serum was assayed by the Bio-Plex assay. **(C)** The level of TNF-α in serum was assayed by the Bio-Plex assay. **(D,E)** Percentage of CD4^+^Foxp3^+^ Tregs in the peripheral blood was assayed by flow cytometry [*n* = 19 per group, ^#^*p* < 0.05 compared with healthy workers (HW); *p < 0.05 compared with subjects under surveillance (SS)]. Error bars indicate mean ± SEM.

To investigate whether Bregs could affect the production of inflammatory cytokines and maintain Tregs in SP, inflammatory cytokines were detected using the Bio-Plex assay, and Tregs in PBMCs were analyzed by flow cytometry. The results showed no significant difference in the serum concentrations of inflammatory cytokines IL-1β, IL-6, and TNF-α between the three groups (Figures 4A–C). The percentage of CD4^+^ T cells expressing Foxp3, which were identified as Tregs, increased significantly in SP (5.92 ± 0.36%) compared with that of SS (3.88 ± 0.38%) and HW (3.91 ± 0.33%) (*p* < 0.001, *p* < 0.001) (Figures 4D,E). These results suggested that Bregs might inhibit the production of inflammatory cytokines and maintain Tregs in SP.

### Th2 Cytokines, Not Th1 Cytokines, Were Increased in SP

**Figure 5:**
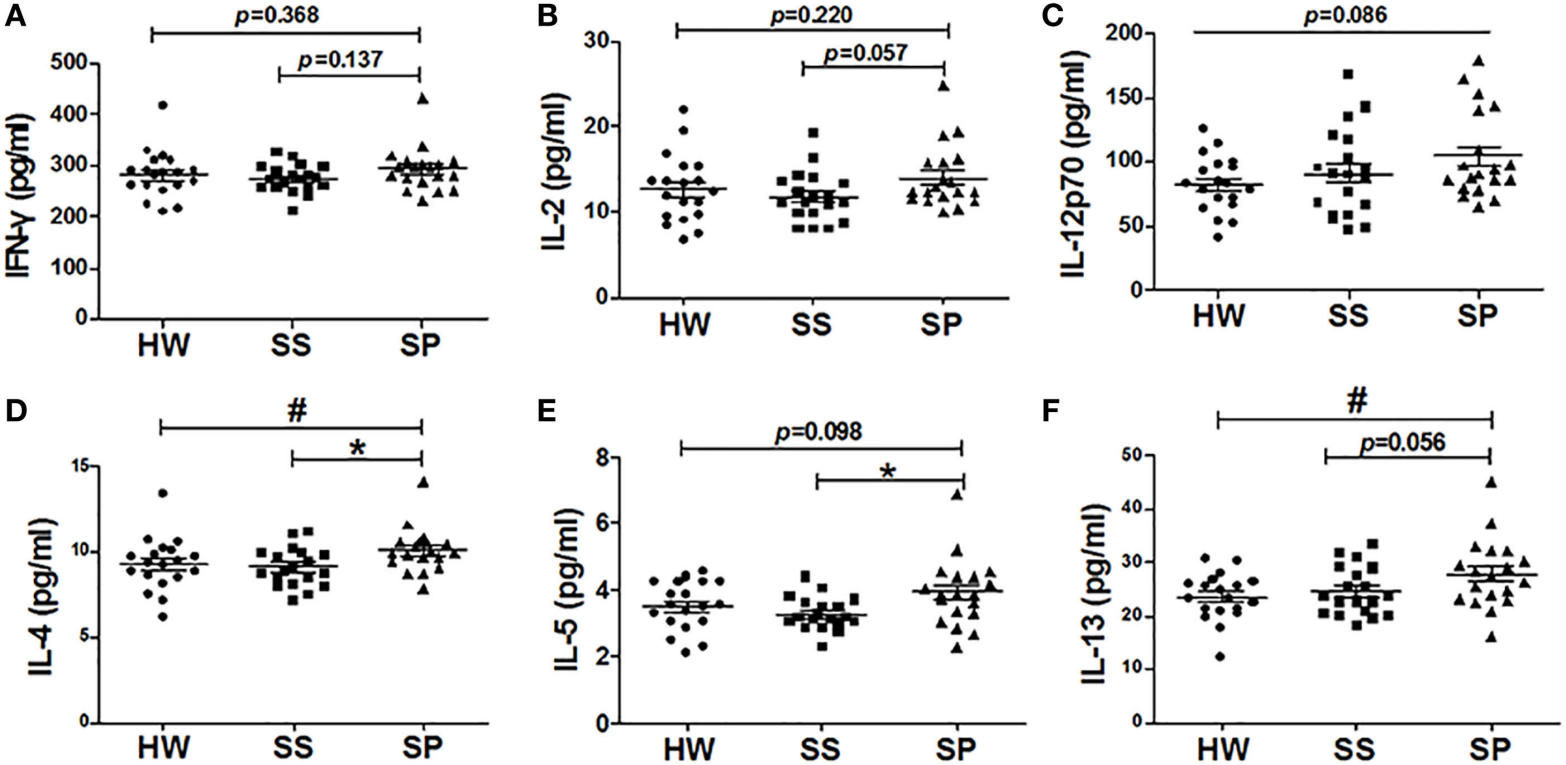
**Levels of Th1/Th2 cytokines were assayed in all participants**. **(A)** The level of IFN-γ in serum was assayed by the Bio-Plex assay. **(B)** The level of IL-2 in serum was assayed by the Bio-Plex assay. **(C)** The level of IL-12 (p70) in serum was assayed by the Bio-Plex assay. **(D)** The level of IL-4 in serum was assayed by the Bio-Plex assay. **(E)** The level of IL-5 in serum was assayed by the Bio-Plex assay. **(F)** The level of IL-13 in serum was assayed by the Bio-Plex assay [*n* = 19 per group, ^#^*p* < 0.05 compared with healthy workers (HW); *p < 0.05 compared with subjects under surveillance (SS)]. Error bars indicate mean ± SEM.

To investigate effects of Bregs on the Th1/Th2 immune response, the levels of Th1/Th2 cytokines were assayed in all participants. There was no significant difference in the levels of Th1 cytokine IFN-γ, IL-2, and IL-12 (p70) in serum between the SP, SS, and HW groups (Figures 5A–C). In contrast, the serum concentration of IL-4 was significantly increased in SP compared to SS and HW (*p* = 0.015, *p* = 0.039) (Figure 5D). The serum concentration of IL-5 was significantly increased in SP compared to SS (*p* = 0.012) and obviously increased compared to HW (*p* = 0.098) (Figure 5E). The serum concentration of IL-13 was significantly increased in SP compared to HW (*p* = 0.011) and obviously increased compared to SS (*p* = 0.056) (Figure 5F). These results suggested that Bregs might inhibit the secretion Th1 cytokines and promote the secretion Th2 cytokines to modulate Th1/Th2 immune responses in SP.

## Discussion

IL-10 is a pleiotropic cytokine produced by a number of leukocyte populations that impacts immune regulation and tissue homeostasis (27, 28). B10s play an important role in maintaining immune homeostasis (29). In this study, we found that the serum concentration of IL-10 was significantly increased in SP compared to SS and HW by protein microarray analysis and the Bio-Plex assay. We compared the frequencies of IL-10-secreting B cells and IL-10-secreting non-B lymphocytes in all participants and determined that the frequency of B10s, not IL-10-producing non-B lymphocytes, was higher in SP than in SS and HW groups.

**Figure 6:**
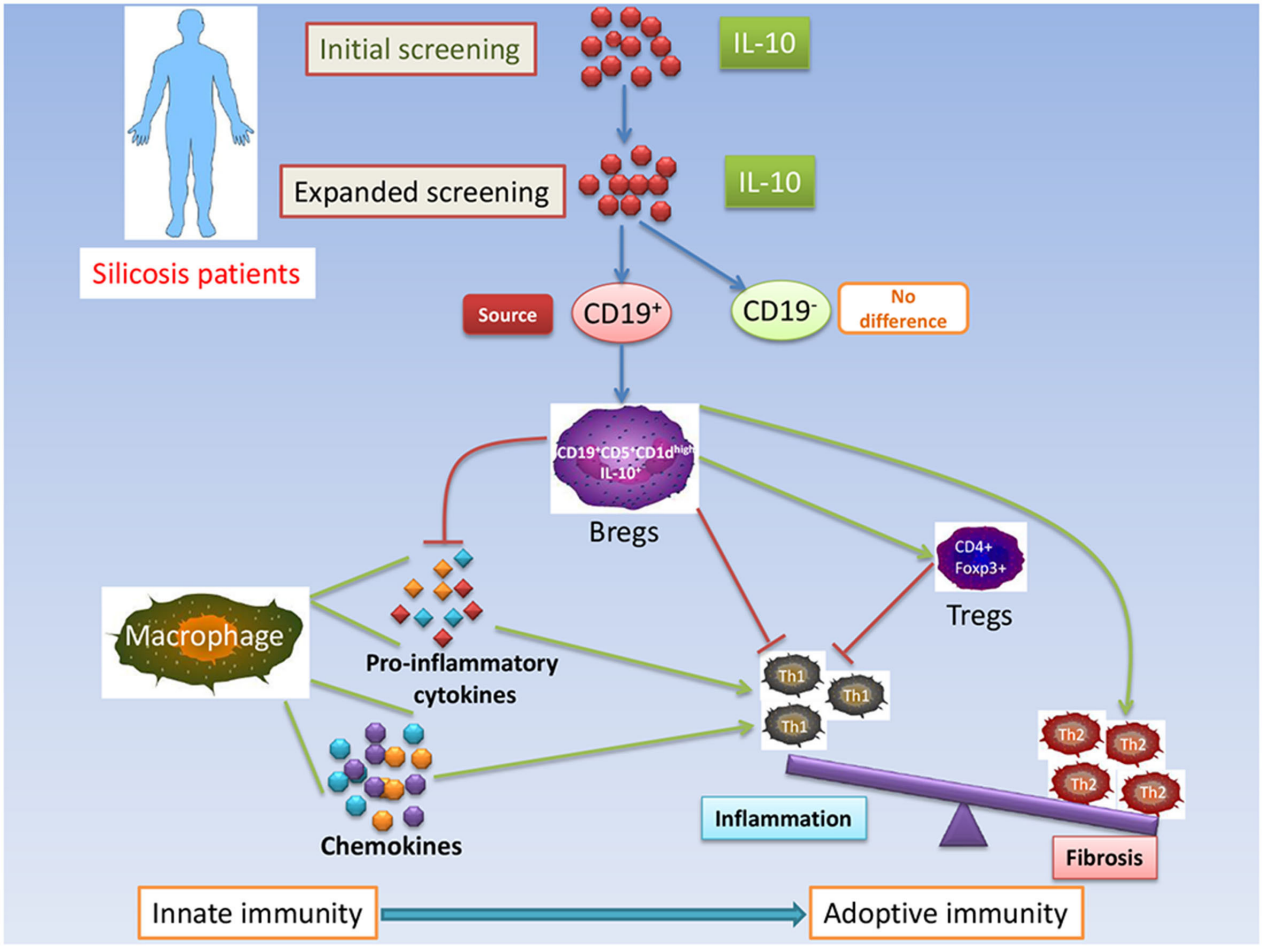
**Schematic representation of IL-10-producing CD1d^high^CD5^+^ regulatory B cells (Bregs) in modulating immune homeostasis in silicosis patients**.

Regulatory B cells are important suppressors of inflammation and other autoimmune responses, producing the negative regulatory cytokines, IL-10 and TGF-β (30). IL-10 and TGF-β-producing B cells mediate tolerance in acute allergic airway inflammation (31). However, IL-10 is considered the hallmark cytokine of Bregs. B10s were the first Bregs to be recognized and were termed “B10” cells (32). Emerging evidence indicates that CD1d^hi^CD5^+^ B cells are responsible for most IL-10 production by B cells (17, 33). The function of Bregs was mediated by IL-10 and dependent on the expression of CD1d (20, 34). A Bregs’ deficiency leads to increased allergic airway Inflammation (35). Our results showed that the percentage of IL-10^+^CD1d^hi^CD5^+^ B cells significantly increased in SP compared to SS and HW groups. The increase of B10s and IL-10^+^CD1d^hi^CD5^+^ B cells in SP might be the results of effective immune response to lung injury. IL-10^+^CD1d^hi^CD5^+^ B cells might play a critical role in modulating immune homeostasis of SP (Figure 6).

The pathogenesis of silicosis involves alveolar cell injury and activation followed by release of cytokines and recruitment of cells into the areas of silica dust deposition (10, 36, 37). Chemokines such as MCP-1, MIP-1, and IP-10 are chemotactic cytokines that recruit specific groups of monocytes/macrophages and lymphocytes, which are involved in many chronic lung diseases (38–41). MCP-1 is a potent chemotactic factor for blood monocytes and produced by various inflammatory cells, which has been considered a potential biomarker for the progress of coal worker’s pneumoconiosis (42). Our results showed that the levels of MCP-1 and IP-10, especially MCP-1, were significantly increased in SS and SP compared to HW. These results suggested that chemokines were involved in the initial stages of silicosis.

It is now widely appreciated that B cells can suppress inflammation and negatively regulate immune responses, in part through the secretion of anti-inflammatory IL-10 (19, 26, 43). The production of pro-inflammatory cytokines, such as IL-1β, IL-6, and TNF-α, by monocytes and macrophages is suppressed by IL-10 (39). In our previous study, we found that the levels of secreted TNF-α and IL-6 proteins in BALF clearly increased in B10-deficiency mice injected with an anti-CD22 antibody (26). Our data revealed that the serum concentrations of IL-1β, IL-6, and TNF-α were not significantly different between the SP, SS, and HW groups. B10s might partly restrain inflammation of silicosis by downregulating the production of pro-inflammatory cytokines. In addition, it has recently been shown that B10s are crucial for the maintenance of Tregs function (44–47). It was reported in mice that tumor-induced B10s could convert resting CD4^+^ non-Tregs into Tregs (48). Another study showed that B10s promoted Tregs accumulation and function by stimulating Foxp3 expression (49). In our previous study, insufficient B10s reduced the frequency of Tregs, whereas Treg depletion did not influence B10 accumulation in silicosis of mice. B10s might act earlier than Tregs during silica-induced lung inflammation and fibrosis (26). Our present data showed that Tregs significantly increased in SP compared to SS and HW. These results indicated that Bregs might maintain Tregs function, which is involved in suppressing inflammation and regulating Th immune responses in SP (Figure 6).

Regulatory B cells have been found to have regulatory properties in inducing primary T cell proliferation and in generating and maintaining CD4^+^ effect T cells (50). It was reported that B cell depletion therapy was associated with changes in T cell repertoire and function in patients with idiopathic thrombocytopenic purpura (51). Our previous studies had demonstrated insufficient B10 could exacerbate Th1 immune response and elevate levels of IFN-γ in silicosis of mice (26). Studies have shown B10s can influence the development of Th2 cells (52, 53). In the current study, the levels of Th1 cytokines IFN-γ, IL-2, and IL-12 (p70) in serum were found to have no significant difference among the three groups. The levels of Th2 cytokines IL-4, IL-5, and IL-13 in serum significantly increased in SP compared to SS and/or HW. These results suggested that increased levels of IL-10/IL-10-producing CD1d^hi^CD5^+^ Bregs might inhibit the production of Th1 cytokines and maintain secretion of Th2 cytokines to regulate the Th1/Th2 balance toward a Th2 phenotype in SP (Figure 6).

The results of the present study show that increased level of inhibitory cytokine IL-10 and number of IL-10-producing CD1d^hi^CD5^+^ Bregs may maintain Tregs and regulate Th1/Th2 polarization, suggesting that IL-10-producing Bregs may play a critical role in modulating immune homeostasis in SP. However, how B cells differentiate into IL-10-producing Bregs and what functions the cells play in silicosis progression remain to be further investigated.

## Author Contributions

JC and YC conceived and designed the research; YC, CL, YL, HZ, WG, BL, JS, and BY performed experiments; YC, CL, FL, DW, and JC analyzed data and interpreted results; YC prepared figures and drafted the manuscript; YC, CL, FL, DW, and JC edited and revised the manuscript; all the authors approved the final manuscript version.

## Conflict of Interest Statement

The authors declare that the research was conducted in the absence of any commercial or financial relationships that could be construed as a potential conflict of interest.
