# Supplementary material for: IL-10-Producing CD1dhiCD5+ Regulatory B Cells May Play a Critical Role in Modulating Immune Homeostasis in Silicosis Patients
Source: Front Immunol. 2017 Feb 13;8:110. doi: 10.3389/fimmu.2017.00110 (PMC5303715; doi:10.3389/fimmu.2017.00110)
Supplement: Supplementary file 1 [file Table_1.DOCX]

| **Table S1. The differential expression proteins between the SP and SS groups were detected by protein microarray** | | |
| --- | --- | --- |
|  | Differences multiples （SP *vs* SS） | *P* value （SP *vs* SS） |
| PDGF-BB | 1.19823 | 0.03795 |
| RANTES | 1.53604 | 0.01801 |
| Nidogen-1 | 1.33996 | 0.01055 |
| P-Cadherin | 1.73360 | 0.02787 |
| TFPI | 1.57759 | 0.00340 |
| TIMP-2 | 1.18382 | 0.03051 |
| MCP-1 | 1.45078 | 0.02256 |
| MIP-1α | 1.71877 | 0.01953 |
| IL-1α | 1.44318 | 0.02886 |
| TNF-β | 1.39074 | 0.00785 |
| Activin A | 3.69698 | 0.04525 |
| TARC | 2.20140 | 0.04439 |
| IL-2 | 1.81557 | 0.00650 |
| GM-CSF | 1.81519 | 0.00885 |
| IL-16 | 1.77328 | 0.04515 |
| Cystatin EM | 2.61670 | 0.04776 |
| IL-15 | 1.61160 | 0.01232 |
| IFN-γ | 1.53379 | 0.02397 |
| IL-7 | 1.41256 | 0.00966 |
| TNF-α | 1.46574 | 0.00656 |
| IL-10 | 1.85954 | 0.00001*** |
| IL-13 | 1.59257 | 0.00881 |
| IL-6 | 1.72179 | 0.00424 |
| IL-5 | 1.62060 | 0.00907 |
| IL-8 | 1.67939 | 0.00875 |
| MIG | 1.69818 | 0.00899 |
| IL-1Rα | 1.43384 | 0.03489 |
| Flt-3L | 1.34865 | 0.04687 |
| Endoglin | 0.72757 | 0.03935 |
| B7-H3 | 0.57586 | 0.01600 |
| TPO | 0.65335 | 0.03879 |
| E-Cadherin | 0.72553 | 0.02856 |
| Axl | 0.67735 | 0.00158 |
| EpCAM | 0.69232 | 0.00214 |
| BMP-7 | 1.42010 | 0.02321 |
| IL-17 | 1.88964 | 0.00539 |
| IL-11 | 2.06385 | 0.00167 |
| IL-12p70 | 1.88203 | 0.00675 |
| OPG | 2.18465 | 0.03143 |
| G-CSF | 2.12616 | 0.00300 |
| I-309 | 2.25109 | 0.01498 |
| MCSF | 1.92857 | 0.02675 |
| IL-12p40 | 2.59837 | 0.03004 |
| BMPR-IA | 0.80932 | 0.00330 |
| IGFBP-5 | 0.76336 | 0.04053 |
| BMP-9 | 1.12549 | 0.04152 |
| SP=silicosis patients (n=5)， SS=subjects under surveillance (n=5)，*** P<0.001 | | |
|  | | |
|  | | |
|  | | |
