# Supplementary material for: IL-10-Producing CD1dhiCD5+ Regulatory B Cells May Play a Critical Role in Modulating Immune Homeostasis in Silicosis Patients
Source: Front Immunol. 2017 Feb 13;8:110. doi: 10.3389/fimmu.2017.00110 (PMC5303715; doi:10.3389/fimmu.2017.00110)
Supplement: Supplementary file 2 [file Table_2.DOCX]

| \| **Table S2. The differential expression proteins between the SP and HW groups were detected by protein microarray** \| \| \| \| --- \| --- \| --- \| \|  \| Differences multiples （SP vs HW） \| *P* value （SP vs HW） \| \| ADAM12 \| 0.60963 \| 0.01172 \| \| Syndecan-3 \| 0.53850 \| 0.03598 \| \| IGF-2R \| 0.67299 \| 0.03206 \| \| Cystatin EM \| 2.99872 \| 0.03672 \| \| IL-11 \| 1.52176 \| 0.04686 \| \| IL-12p70 \| 1.54385 \| 0.02675 \| \| Axl \| 0.57159 \| 0.03268 \| \| Resistin \| 0.61844 \| 0.04148 \| \| IL-10 Rb \| 0.58832 \| 0.02872 \| \| CD40L \| 0.66370 \| 0.02760 \| \| IL-17R \| 0.43349 \| 0.01555 \| \| GM-CSF \| 1.52569 \| 0.01937 \| \| IL-10 \| 1.53560 \| 0.00082*** \| \| IL-6 \| 1.37028 \| 0.03692 \| \| Endoglin \| 0.56321 \| 0.02026 \| \| HCC-4 \| 0.73645 \| 0.00650 \| \| HCC-1 \| 0.89030 \| 0.04479 \| \| Serpin A4 \| 1.52774 \| 0.00353 \| \| SP=silicosis patients (n=5)，HW=healthy workers with exposure to silica dust (n=5)，*** P<0.001 \| \| \| |
| --- | --- | --- | --- | --- | --- | --- | --- | --- | --- | --- | --- | --- | --- | --- | --- | --- | --- | --- | --- | --- | --- | --- | --- | --- | --- | --- | --- | --- | --- | --- | --- | --- | --- | --- | --- | --- | --- | --- | --- | --- | --- | --- | --- | --- | --- | --- | --- | --- | --- | --- | --- | --- | --- | --- | --- | --- | --- | --- | --- | --- | --- | --- | --- |
|  |
|  |
