# Supplementary material for: IL-10-Producing CD1dhiCD5+ Regulatory B Cells May Play a Critical Role in Modulating Immune Homeostasis in Silicosis Patients
Source: Front Immunol. 2017 Feb 13;8:110. doi: 10.3389/fimmu.2017.00110 (PMC5303715; doi:10.3389/fimmu.2017.00110)
Supplement: Supplementary file 3 [file Table_3.DOCX]

| **Table S3. The differential expression proteins in serum of HW, SS and SP were detected by protein microarray** | | | | |
| --- | --- | --- | --- | --- |
|  | Mean Fluorescence Intensity | | | *P* value |
|  | HW ( n=5) | SS ( n=5) | SP( n=5) |  |
| PDGF-BB | 448763.30±34891.93 | 394266.43±21409.18 | 472422.09±23077.79 | 0.0683 |
| RANTES | 292974.30±27851.24 | 239392.99±25326.73 | 367716.57±35097.49 | 0.0512 |
| Nidogen-1 | 189942.02±13586.88 | 169996.97±10394.16 | 227788.92±13965.38 | 0.3305 |
| P-Cadherin | 122437.04±18555.13 | 100657.10±14913.77 | 174498.99±23152.38 | 0.0191* |
| TFPI | 95760.82±8764.83 | 69364.22±4733.15 | 109428.05±8524.44 | 0.0532 |
| TIMP-2 | 50421.34±4656.82 | 45389.08±2508.68 | 53732.58±1955.85 | 0.2301 |
| MCP-1 | 46180.13±4834.31 | 31494.74±2680.80 | 45691.99±4265.46 | 0.0395* |
| MIP-1α | 33494.32±6198.28 | 16856.31±2087.50 | 28972.16±3599.20 | 0.0471* |
| IL-1α | 20213.13±2966.88 | 14061.46±786.33 | 20293.26±2208.06 | 0.0144* |
| TNF-β | 15793.42±773.84 | 14121.98±250.96 | 19640.07±1547.60 | 0.0066** |
| Activin A | 12258.16±8927.56 | 3369.53±1002.77 | 12457.10±3700.96 | 0.4492 |
| TARC | 8276.34±1627.61 | 5880.86±1371.76 | 12946.15±2629.52 | 0.0683 |
| IL-2 | 9023.07±1698.74 | 5830.51±359.37 | 10585.72±1252.32 | 0.0512 |
| GM-CSF | 6582.32±885.43 | 5532.52±383.27 | 10042.58±789.23 | 0.0021** |
| IL-16 | 7426.00±2577.55 | 4274.17±144.69 | 7579.31±1386.23 | 0.3305 |
| Cystatin EM | 2957.34±554.86 | 3389.09±489.94 | 8868.23±2294.50 | 0.0191* |
| IL-15 | 12624.88±1813.86 | 9106.89±243.46 | 14676.65±1715.02 | 0.0532 |
| IFN-γ | 12206.40±1440.40 | 9166.63±464.02 | 14059.65±1698.58 | 0.0620 |
| IL-7 | 11891.65±860.89 | 9612.41±563.88 | 13578.11±1029.51 | 0.0191* |
| TNF-α | 10419.43±657.73 | 9154.29±294.20 | 13417.84±1132.59 | 0.0062** |
| IL-10 | 8819.15±688.10 | 7282.81±263.69 | 13542.66±592.11 | 0.000009*** |
| IL-13 | 10816.94±931.92 | 7998.53±446.87 | 12738.21±1303.04 | 0.0144* |
| IL-6 | 9883.33±591.35 | 7865.59±522.95 | 13542.90±1338.76 | 0.0025** |
| IL-5 | 10549.09±1074.20 | 7736.41±575.61 | 12537.63±1279.99 | 0.0193* |
| IL-8 | 8838.66±799.10 | 6939.75±389.60 | 11654.54±1311.61 | 0.011* |
| MIG | 10248.55±1770.79 | 6808.05±329.19 | 11561.31±1347.35 | 0.0606 |
| IL-1Rα | 11181.03±1939.45 | 7431.09±455.14 | 10655.02±1186.61 | 0.1427 |
| Flt-3L | 12384.12±1721.32 | 8466.63±697.09 | 11418.53±1046.61 | 0.104 |
| Endoglin | 17445.51±2582.20 | 13504.45±1394.02 | 9825.47±542.34 | 0.0279* |
| B7-H3 | 8024.78±738.28 | 10781.18±1383.63 | 6208.50±586.83 | 0.0185* |
| TPO | 9775.12±3660.86 | 6152.31±709.14 | 4019.58±493.38 | 0.2079 |
| E-Cadherin | 10587.38±3924.38 | 5879.63±502.93 | 4265.88±337.09 | 0.1711 |
| Axl | 4756.02±782.01 | 4013.44±252.46 | 2718.50±112.79 | 0.0322 |
| EpCAM | 6856.58±3035.95 | 3348.09±161.32 | 2317.96±165.98 | 0.2021 |
| BMP-7 | 4749.55±677.31 | 2677.58±164.69 | 3802.43±366.48 | 0.0236* |
| IL-17 | 3489.07±476.56 | 2655.60±216.65 | 5018.13±586.22 | 0.0098** |
| IL-11 | 3387.11±527.66 | 2497.46±198.91 | 5154.37±536.96 | 0.0040** |
| IL-12p70 | 3622.44±323.16 | 2971.53±313.69 | 5592.51±651.84 | 0.0043** |
| OPG | 2971.35±721.28 | 2220.63±118.62 | 4851.29±1003.35 | 0.0605 |
| G-CSF | 3451.83±1068.43 | 1895.28±172.79 | 4029.68±477.94 | 0.1144 |
| I-309 | 4009.96±2133.20 | 1303.31±308.43 | 2933.86±429.00 | 0.3482 |
| MCSF | 3270.81±1334.72 | 1517.01±178.66 | 2925.65±488.57 | 0.3181 |
| IL-12p40 | 8351.64±6411.80 | 1342.83±232.33 | 3489.18±781.39 | 0.4227 |
| BMPR-IA | 1565.98±53.10 | 1839.92±61.07 | 1489.08±59.05 | 0.0026** |
| IGFBP-5 | 2683.77±573.87 | 1794.68±137.72 | 1369.99±106.37 | 0.0543 |
| BMP-9 | 1001.56±119.74 | 996.00±36.33 | 1120.98±36.56 | 0.4398 |
| ADAM12 | 1611.66±182.48 | 1110.04±142.61 | 982.52±64.80 | 0.0178* |
| Syndecan-3 | 1849.03±276.68 | 1162.10±40.92 | 995.70±195.95 | 0.0228* |
| IGF-2R | 2223.52±197.70 | 1957.38±204.79 | 1496.41±199.14 | 0.0692 |
| Resistin | 8012.43±1152.42 | 6146.85±780.05 | 4955.19±510.35 | 0.0749 |
| IL-10 Rb | 6936.38±1023.98 | 5502.60±775.35 | 4080.84±319.61 | 0.0638 |
| CD40L | 5508.73±468.15 | 5354.54±542.77 | 3656.16±505.95 | 0.0435* |
| IL-17R | 8098.39±1426.83 | 6793.84±2216.52 | 3510.53±457.98 | 0.1385 |
| HCC-4 | 38493.92±1586.72 | 33087.46±1713.57 | 28348.66±2282.66 | 0.0086** |
| HCC-1 | 35745.68±1384.78 | 32676.73±1505.74 | 31824.34±897.16 | 0.1188 |
| Serpin A4 | 28257.21±1392.83 | 35370.14±2023.84 | 43169.60±3378.16 | 0.0033** |
| HW=healthy workers with exposure to silica dust， SS=subjects under surveillance，SP=silicosis patients, Values are expressed as means±SEM, * P<0.05, ** P<0.01, *** P<0.001 | | | | |
|  | | | | |
