# Supplementary material for: IL-10-Producing CD1dhiCD5+ Regulatory B Cells May Play a Critical Role in Modulating Immune Homeostasis in Silicosis Patients
Source: Front Immunol. 2017 Feb 13;8:110. doi: 10.3389/fimmu.2017.00110 (PMC5303715; doi:10.3389/fimmu.2017.00110)
Supplement: Supplementary file 4 [file Table_4.DOCX]

| **Table S4. Serum protein levels were measured by the Bio-Plex assay in all participants** | | | | |
| --- | --- | --- | --- | --- |
| (pg/ml) | HW( n=19) | SS( n=19) | SP( n=19) | *P* value |
| IL-1β | 2.58±0.17 | 2.54±0.15 | 2.57±0.14 | 0.985 |
| IL-1ra | 398.37±23.68 | 366.49±16.46 | 429.10±23.86 | 0.132 |
| IL-2 | 12.65±0.91 | 11.83±0.67 | 14.08±0.86 | 0.154 |
| IL-4 | 9.25±0.35 | 9.08±0.26 | 10.15±0.28 | 0.033* |
| IL-5 | 3.50±0.17 | 3.27±0.11 | 3.93±0.23 | 0.038* |
| IL-6 | 7.93±0.43 | 7.53±0.36 | 8.69±0.67 | 0.262 |
| IL-7 | 17.24±0.66 | 16.77±0.62 | 18.29±0.65 | 0.243 |
| IL-8 | 73.01±19.15 | 55.28±7.34 | 36.60±1.43 | 0.106 |
| IL-9 | 61.03±4.45 | 54.00±2.70 | 66.03±4.18 | 0.089 |
| IL-10 | 12.32**±**0.82 | 12.67**±**0.90 | 15.97**±**1.34 | 0.031* |
| IL-12(p70) | 82.26**±**5.02 | 91.14**±**7.80 | 104.63**±**7.89 | 0.086 |
| IL-13 | 23.53**±**1.02 | 24.67**±**1.04 | 27.99**±**1.49 | 0.031* |
| IL-15 | ND | ND | ND |  |
| IL-17 | 149.59±6.01 | 151.80±3.96 | 156.85±5.01 | 0.585 |
| IFN-γ | 282.19**±**10.62 | 274.42**±**6.33 | 293.93**±**9.87 | 0.323 |
| TNF-α | 41.84**±**2.92 | 38.58**±**1.77 | 45.43**±**3.37 | 0.225 |
| Eotaxin | 387.67±43.74 | 356.28±19.30 | 422.55±34.76 | 0.396 |
| FGF basic | 23.86±1.15 | 22.17±0.41 | 25.08±0.88 | 0.070 |
| G-CSF | 109.28±5.45 | 98.51±3.45 | 120.32±6.23 | 0.016* |
| GM-CSF | 41.19±2.83 | 37.11±1.73 | 41.72±2.02 | 0.289 |
| PDGF-BB | 4001.09±267.84 | 4127.99±361.15 | 4382.13±283.44 | 0.672 |
| RANTES | 2988.26±95.94 | 3149.74±96.2 | 2971.82±84.72 | 0.33 |
| VEGF | 108.83±8.50 | 113.33±12.66 | 133.84±13.98 | 0.296 |
| IP-10 | 1366.78**±**94.14 | 1787.52**±**175.03 | 1783.62**±**185.09 | 0.102 |
| MCP-1 | 41.56**±**1.88 | 51.01**±**2.36 | 52.61**±**2.95 | 0.004* |
| MIP-1α | 6.45**±**0.75 | 8.34**±**1.87 | 5.13**±**0.15 | 0.160 |
| MIP-1β | 169.79**±**17.71 | 196.89**±**16.32 | 150.39**±**15.35 | 0.148 |
| HW=healthy workers with exposure to silica dust, SS=subjects under surveillance, SP=silicosis patients，“ND” indicates not detected; * P<0.05 | | | | |
